# Working alliance and its link to guidance in an internet-based intervention for depressive disorders: a secondary analysis of a randomized controlled trial

**DOI:** 10.3389/fpsyt.2024.1448823

**Published:** 2024-09-11

**Authors:** Raoul Haaf, Cora Schefft, Rico Krämer, Jan Philipp Klein, Stephan Köhler

**Affiliations:** ^1^ Department of Psychiatry and Psychotherapy, Charité - Universitätsmedizin Berlin, Corporate Member of Freie Universität Berlin and Humboldt- Universität zu Berlin, Berlin, Germany; ^2^ Department of Psychiatry, Psychosomatics and Psychotherapy, University of Lübeck, Lübeck, Germany; ^3^ Center for Brain, Behavior and Metabolism, University of Lübeck, Lübeck, Germany

**Keywords:** internet-based intervention, depression, therapeutic alliance, adherence, guidance

## Abstract

**Background:**

Guided Internet-based interventions (IBIs) are typically found to be more effective than unguided ones, but the reasons behind this are not well understood. The therapist-client working alliance, crucial in face-to-face psychotherapy, is also increasingly recognized as an important factor in IBIs. This study examines trajectories of the working alliance and its relationship to therapeutic guidance through a secondary analysis of a randomized controlled trial (RCT) on Selfapy, a 12-week IBI based on cognitive behavioral therapy for depressive disorders. The trial compared a therapist-guided version (with weekly calls) to an unguided version (n = 301, mean age 37 years, 83% female, mean BDI-II = 30.09).

**Methods:**

Based on an intention-to-treat approach, this study investigates within- and between-group differences in the quality of the working alliance, assessed with the WAI-SR questionnaire at mid- and post-treatment via repeated measures ANOVA. Furthermore, correlations and mediation analyses were conducted to explore the relationship between the working alliance and outcomes, as well as adherence parameters.

**Results:**

Findings indicate that the IBI was successful in fostering a robust working alliance in both intervention groups, with similar ratings at mid-treatment but significantly higher ratings in the guided group at post-treatment (Cohen’s d = -0.38). Post-treatment working alliance scores were positively linked to symptom reduction at post-treatment (guided: r = .25, unguided r = .15) and follow-up (guided: r = .25, unguided: r = .17). In the unguided group, the association was primarily driven by the subscale task. Serial mediation analysis indicated that the relationship between guidance and outcomes at follow-up was mediated by working alliance (b = 0.59; 95% CI: 0.14, 1.22) and a link between working alliance and adherence (b = 0.15; 95% CI: 0.04, 0.34).

**Conclusions:**

Considering limitations like using a questionnaire developed for face-to-face therapy, findings support the importance of the working alliance in guided IBIs, while also providing new insights into its role and formation in unguided IBIs. The potential benefits of a strong working alliance, notably by improving adherence, may prove crucial for integrating guided as well as unguided IBIs into routine use, indicating the need for additional research in this context.

**Clinical Trial Registration:**

https://tinyurl.com/2p9h5hnx, German Clinical Trials Register DRKS00017191.

## 1 Introduction

Internet-based interventions (IBIs) have demonstrated to be effective in treating depressive symptoms and provide a low-cost, low-threshold alternative to face-to-face (f2f) psychotherapy (1, 2). However, the factors influencing and mediating treatment success in IBIs are less clear (3). Many IBIs have used some form of guidance, i.e. additional human support through communication via email, telephone, or video which has been linked to increased adherence and more favourable outcomes compared to unguided interventions (2, 4, 5). Many uncertainties remain however regarding the factors that mediate the potential positive effects of guidance (4, 6). The working alliance has long been identified with strong evidence as one of the main mediators of treatment success in f2f therapy (7–9) and is therefore also increasingly in focus of research on IBIs. In particular, two recent studies indicated a close link of the working alliance to guidance (10, 11).

A widely accepted concept of the working alliance is the one suggested by Bordin (12), which describes the relationship between client and therapist in terms of three dimensions: the emotional *bond* aspect of the relationship, and their agreement on therapy *tasks* and *goals.* Although there has been concern regarding the implementation of a functioning working alliance in IBIs due to the absence of f2f communication, studies have reported alliance ratings in guided (13), as well as unguided IBIs (14) to be comparable to those in f2f therapies. Notably, blended Cognitive Behavioural Therapy (bCBT), which combines IBIs with traditional f2f therapy, has been reported to yield significantly higher working alliance scores compared to both treatment as usual and f2f therapy alone (15). Further supporting the hypothesis that the working alliance in IBIs may be of similar importance as in f2f therapy, several meta-analyses have reported a close link to outcomes (16–18) and adherence in IBIs (4, 10). However, differences in these aspects due to the presence or absence of guidance in IBIs, have not been thoroughly investigated thus far (11). Furthermore, the underlying mechanisms of a strong working alliance with regard to Bordin’s (12) dimensions *goals, bond, tasks* in IBIs remain ambiguous. Several studies suggest that agreement on therapy *goals* and *tasks* may be of particular significance for the working alliance in IBIs and may also be critical for treatment outcomes (13, 18). Supporting this, benefits of digital therapy elements on the *goals* and *tasks* dimensions have also been highlighted for blended therapy (15), although conflicting evidence exists regarding the *goals* dimension (19). Research on the role of guidance in IBIs in this regard is still limited. Bur et al. (10) found that guidance was linked to higher working alliance scores, primarily due to higher ratings in the *bond* subscale. Conversely, only the *goals* and *tasks* subscales correlated with a reduction in depressive symptoms at post-treatment. Comparing varying degrees of guidance, Luo et al. (11) reported ratings of the working alliance to be linked to outcomes only in a video-supported condition, but they did not report on the individual dimensions of the working alliance.

A better understanding of the role of the working alliance in the therapeutic process of IBIs in the presence or absence of guidance could be essential to optimize both guided and unguided IBIs. In the present study we therefore examined the working alliance as part of a secondary analysis of a RCT (randomized controlled trial) on the efficacy of Selfapy, an IBI in form of an iCBT (Internet-Based Cognitive Behavioural Therapy) for mild to severe depression. While the primary study examined efficacy of the intervention in three groups: (1) a therapist-guided, (2) an unguided version of *Selfapy* and (3) a control group which had no access to the IBI and merely received weekly standardized mindfulness exercises via email, our focus in the present study was on the guided and unguided intervention groups only. Our objectives were threefold: Firstly, we examined between and within-group trajectories and effect sizes of the working alliance, expecting generally higher ratings in the guided group. Secondly, we explored associations between the working alliance, reduction of depressive symptoms, and adherence. Thirdly, we further investigated the relationship between guidance and outcomes, hypothesizing that the working alliance and adherence would mediate this relationship.

## 2 Methods

### 2.1 Data collection

Data for the current investigation came from a previously published RCT on the efficacy of the IBI *Selfapy* (20, 21), which was approved by the ethics committee of the medical faculty of the Charité University Medicine Berlin and was conducted in line with the Helsinki Declaration of 1975, as revised in 2008. A more detailed account on the study’s rationale, the intervention and its methods are available in the published protocol and the published primary outcomes (20, 21). Participants with depressive symptoms were recruited via the providers website (www.selfapy.de), advertisements in social media and information brochures from health-insurance companies. Potential participants were screened by telephone by trained interviewers (psychologists and medical students) using the MINI International Neuropsychiatric Interview (22). Inclusion criteria were (1) age between 18 and 65 years; (2) sufficient knowledge of German to use and understand the IBI; (3) reliable Internet access; (4) a Beck Depression Inventory (BDI-II) (23) score ≥13; (5) willingness to provide electronic data; and (6) diagnosis of major depressive disorder or dysthymia based on the MINI, in accordance with the International Statistical Classification of Diseases tenth revision (ICD-10: F32, F33, F34). Exclusion criteria were (1) diagnoses of a bipolar disorder or schizophrenia; (2) acute psychotic symptoms; (3) current substance dependence (within the past 6 months) or withdrawal syndrome (ICD-10: F1x2, F1x3); (4) acute suicidality. The recruitment took place throughout all of Germany. Participants were randomly assigned to one of three groups (guided, unguided, control) in a ratio of 3:3:2.

### 2.2 Intervention

A more detailed description of the IBI *Selfapy* has been reported elsewhere (20, 21, 24). Briefly, the intervention consists of six core modules and six additional optional in-depth modules based on cognitive behavioral therapy (CBT). Duration of the intervention is twelve weeks. To meet the reality of care, participants were not influenced nor advised to change their existing treatment patterns and were free to seek pharmacological or psychological treatments. In the unguided group the participants carried out the program independently but had access to a chat functionality that allowed them to ask questions regarding the correct use of the course. In the guided group, participants received additional personal therapeutic guidance by a psychotherapist-in-training for the entire duration of the program through weekly telephone calls of 25–30 minutes duration. The content of the individual conversations was based on the course content (20) and included reflecting on the weekly topic and addressing personal resources and behavioural activation.

### 2.3 Measures

Depressive symptoms were assessed using the BDI-II (Beck Depression Inventory-II; (23) at pre-treatment (T1), 6 weeks after pre-treatment (T2; mid-treatment), 12 weeks after pre-treatment (T3; post-treatment) and 24 weeks after pre-treatment (T4; follow-up). The primary outcome measure was the change of depressive symptoms at post-treatment (Δ BDI-II T1-T3). Participants who did not complete post-treatment resp. follow-up questionnaires were considered dropouts. The quality of the working alliance was assessed by the German version the WAI-SR [Working alliance inventory- short revised (25)] at mid-treatment (T2) and post-treatment (T3). The WAI-SR is a 12-item self-report questionnaire and encompasses three subscales, which represent the three dimensions of Bordin’s theory of the therapeutic alliance: *goal*, *task*, *bond* (12). The *goal* subscale indicates the extent to which the patient agrees with the therapy goals and the underlying principles for achieving them. The subscale *task* refers to the agreement on concrete tasks for patient and therapist with regard to the therapeutic techniques used. The *bond* subscale represents the relationship between therapist and patient based on a certain level of trust and attachment between the therapeutic partners. Subscale scores (four items each) and total score are calculated as the mean ranging from 1 to 5. The wording of the questionnaire was the same for both groups. Examples: Bond subscale: ‘My therapist and I respect each other.’ [WAI-SR-Item 5]. Tasks subscale: ‘What I do in therapy opens up new perspectives on my problem.’ [WAI-SR-Item 2]. Goals subscale: ‘My therapist and I work together to set therapy goals.’ [WAI-SR-Item 4]. Participants were asked to evaluate their overall therapy program. The guided group did not receive additional instructions to specifically rate their human therapeutic interactions or the IBI. An internal consistency of the WAI-SR for outpatient and inpatient samples of *α* > .80 and a convergent validity with the Helping Alliance Questionnaire (26, 27) of r > .64 has been reported (25). The internal consistency for the current sample at each assessment was Cronbach’s α = .95 -.96 for all participants and α = .90 -.97 for the individual groups.

Two parameters were used to quantify adherence: (1) the parameter *module progress* captured the completion of the six core and six optional modules as a continuous variable ranging from 0 to 12; (2) the parameter *course completion* captured the completion of the six core modules as a dichotomous variable (y/n).

### 2.4 Statistical analyses

Analyses were performed in SPSS28 (IBM Corp. 2021) and R (version 4.1.1.). Analyses were based on an intention-to-treat (ITT) principle, missing values in the data were replaced using Multiple Imputation by Chained Equations (MICE; with m = 5 imputations). The results reported in the text refer to the MICE ITT analyses unless specified otherwise.

First, a repeated measures ANOVA was conducted at an alpha level of α = .05, including both assessment times of the working alliance (T2, T3), to evaluate the effects of *group*, *time*, and *group x time* interaction for the WAI-SR total score and subscale scores. *Post-hoc* pairwise comparisons were conducted to further investigate differences between groups at T2 and T3. To counteract bias due to multiple testing, Bonferroni correction was applied. Within and between group effect sizes are reported as Cohen’s d (d ≈ 0.2 small, d ≈ 0.5 medium, d ≥ 0.8 large effect size) (28).

Second, we sought to investigate potential associations of the quality of the working alliance with treatment outcome and adherence parameters. For the association of the working alliance with outcomes, separate partial correlations were calculated between WAI-SR scores at mid- and post-treatment with outcomes at post-treatment (Δ BDI-II T1-T3) and at follow-up (Δ BDI-II T1-T4). For the association of working alliance and adherence parameters, partial correlations were calculated between WAI-SR scores and *module progress* at post-treatment, as well as binary logistic regressions for the dichotomous adherence parameter *course completion*. The BDI-II score at pre-treatment was included as a control variable in each analysis.

**Figure 1:**
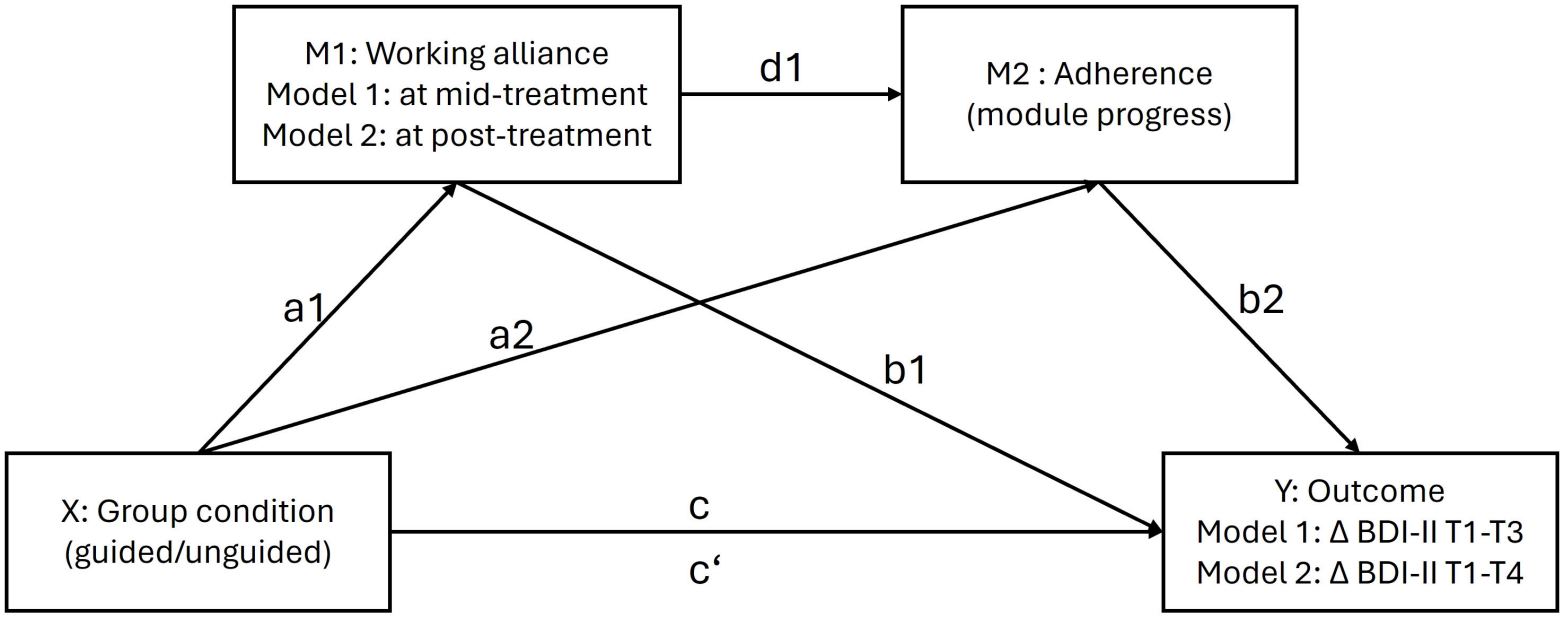
Hypothesized serial mediation model with working alliance (a1*b1) and a link between working alliance and adherence (a1*d1*b2) mediating the relationship between guidance and outcomes. M1 = mediator 1; M2 = mediator 2; X = independent variable; Y = dependent variable. Lower case letters indicate direct associations between variables. all analyses were corrected for pre-treatment depression scores.

Third, drawing on previous research (10, 29) we conducted two separate serial mediation analyses to test the hypothesis that the relationship between guidance and outcomes would be mediated by (1) working alliance, (2) adherence and (3) a link between working alliance and adherence (**Figure 1**). This approach follows the modern Hayes and Rockwood (30) framework of mediation, which unlike the traditional mediation framework (31), emphasizes that mediation through an indirect effect can be assumed even if there is no significant direct or total effect. Following recommendations with regard to the timeline of mediator and outcome effect (32), model 1 tested whether the relationship between guidance and outcomes at post-treatment was mediated by working alliance at mid-treatment and adherence. Model 2 tested whether the relationship between guidance and outcomes at follow-up was mediated by working alliance at post-treatment and adherence. To this end a serial mediation model (PROCESS, model 6; (33)) was adopted using the standard bootstrapping method with 5000 samples to construct a 95% confidence interval of mediating effects. Point estimates of indirect effects are considered statistically significant if zero is not included in the 95% confidence interval. Only continuous variables are allowed as mediators in this model, and therefore only *module progress* could be studied as an adherence parameter here. The group condition (guided vs unguided) was the independent variable (X), Δ BDI-II at post-treatment (model 1) and at follow-up (model 2) was the dependent variable (Y), the first mediator was total WAI-SR score at mid-treatment (M1; model 1) and post-treatment (M1; model 2), the second mediator was *module progress* (M2). The BDI-II score at pre-treatment was included as a control variable in all analyses. Significant models were rerun additionally correcting for prior change in depressive symptoms.

## 3 Results

### 3.1 Participants

817 prospective participants expressed interest in the study, of which 322 withdrew before the inclusion interview. Of the remaining 495 interviewees, 94 did not meet inclusion criteria while 401 participants were included in the primary study (Flowchart reported in 20). In the present study only the 301 participants in the intervention groups were analyzed (guided group: n = 151, unguided group: n = 150). Upon study entrance 91.4% of included participants fulfilled the diagnostic criteria for a current major depressive episode and 8.6% for dysthymia in accordance with the International Statistical Classification of Diseases tenth revision (ICD-10: F32, F33, F34). Data at baseline indicated an average mild-to-severe level of depression in all participants (mean BDI-II = 32.3, score range 13-56). The average age of participants was 37.4 ± 10.7 years and the sample included 252 (83.7%) females. Further baseline characteristics of the sample are reported in **Table 1**.

**Table 1:** Pre-treatment demographics for the guided and unguided group.

Characteristic	Guided, N=151	Unguided, N=150	Total sample, n=301
Sex, n (%)			
Female	126 (83.4)	126 (84.0)	252 (83.7)
Male	25 (16.6)	24 (16.0)	49 (16.3)
**Age, mean (SD)**	38 (10.7)	37 (10.8)	37.37 (10.7)
Relationship status, n (%)			
Married/ partnered	54 (35.8)	33 (22.0)	87 (28.9)
No partner (divorced, separated, widowed)	19 (12.6)	8 (5.3)	27 (8.9)
Single	68 (45.0)	75 (50.0)	143 (47.5)
Not reported	10 (6.6)	34 (22.7)	44 (14.6)
Children, n (%)			
Yes	31 (20.5)	33 (22.0)	64 (21.3)
No	89 (58.9)	99 (66.0)	188 (62.5)
Not reported	31 (20.5)	18 (12.0)	49 (16.3)
Professional training, n (%)			
Still in training	11 (7.3)	6 (4.0)	17 (5.6)
Apprenticeship	28 (18.5)	19 (12.7)	47 (15.6)
Advanced vocational training	17 (11.3)	15 (10.0)	32 (10.6)
College or post-graduate degree	39 (25.8)	45 (30.0)	84 (27.9)
No training	15 (9.9)	18 (12.0)	33 (11.0)
Other	1 (0.7)	0 (0.0)	1 (0.3)
Not reported	40 (26.5)	47 (31.3)	87 (28.9)
Employment, n (%)			
Yes	85 (56)	90 (60)	175 (58.1)
In school/training	12 (8)	6 (4)	18 (6.0)
No/other	7 (4.6)	3 (2)	10 (3.3)
Not reported	47 (31)	51 (34)	98 (32.6)
Current psychotherapy at 12 weeks, n (%)			
Yes	10 (6.6)	12 (8.0)	22 (7.3)
No	120 (79.5)	104 (69.3)	224 (47.4)
Not reported	21 (13.9)	34 (22.7)	55 (18.3)
Start of psychotherapy during intervention, n (%)			
Yes	6 (4.0)	5 (3.3)	11 (3.7)
No	124 (82.1)	111 (74.0)	235 (18.3)
Not reported	21 (13.9)	34 (22.7)	55 (24.9)
Antidepressants at baseline, n (%)			
Yes	45 (29.8)	25 (16.7)	70 (23.3)
No	106 (70.2)	125 (83.3)	231 (76.7)
Not reported	0 (0.0)	0 (0.0)	0 (0)
Antidepressants at 12 weeks, n (%)			
Yes	26 (17.2)	40 (26.7)	66 (21.9)
No	125 (82.8)	110 (73.3)	235 (87.1)
Not reported	0 (0.0)	0 (0.0)	0 (0)
Current major depressive episode, n (%)			
Yes	143 (95)	132 (88)	275 (91.4)
No	8 (5)	18 (12)	26 (8.6)
Lifetime major depressive episode n (%)			
Yes	94 (62)	103 (69)	197 (65.4)
No	57 (38)	47 (31)	104 (34.6)

### 3.2 Intervention outcomes

Using Kolmogorov-Smirnov tests, no violation of the normal distribution was identified for any of the measures. Results of the RCT on the efficacy of the IBI have been previously reported (20). In brief, within group effect sizes for BDI-II at posttreatment were large for both the guided (d = 1.44, 95%CI: [1.21; 1.68]) and unguided (d = 1.38, 95%CI: [1.15; 1.65]) group, whereas the control group (waiting list) showed no effect (d = 0.07, 95%CI: [-0.21; 0.37]). Between group effect sizes compared to controls were large in both intervention groups, whereas the effect size between the guided and unguided group was negligible (d = 0.20, 95%CI: [-0.04; 0.45]). Follow-up assessments showed that the treatment effects were maintained to a certain level, with no significant group differences between the intervention groups.

### 3.3 Dropouts and adherence parameters

The overall dropout rate at post-treatment was 17.6%. Group-comparison using a t-statistic revealed a lower drop-out rate in the guided (12.6%) compared to the unguided group (22.7%) (t(299) = 3.21, p = .022). The overall dropout rate at follow-up was 57.8%. Group comparison of dropouts at follow-up indicated no statistical difference (t(299) = 0.3, p = .76). Little’s MCAR test resulted in χ2 = 39.93 (df = 31, p >.05), indicating that data was missing at random. Descriptive results on means and standard deviations of the measured adherence parameters across time are reported in **Table 2**. A mean of 9.35 (SD = 2.3) modules were completed by each participant in the intervention groups. The intervention groups did not differ significantly regarding module progress (t(299) = 0.60, p = .55). 255 (84.7%) participants from the intervention groups completed the main course (six core modules). The intervention groups did not differ significantly regarding course completion (t(299) = 0.62, p = .54). As previously reported elsewhere (24) the median number of messages sent via chat did not differ between groups (both median = 4, interquartile range (IQR)_guided_: 1–10; IQR_unguided_: 0–13, T = 9852, p = .98). However, the groups varied in the number of words per message (median_guided_ = 732, IQR: 386–2149.5; median_unguided_ = 1484, IQR: 538–5829; T = 7528, p = .001). On average, participants in the guided group had 8 calls (SD = 3.6) during the intervention, with an average duration of 22 minutes (SD = 6.5).

**Table 2:** Means and standard deviations of depressive symptoms (BDI-II), working alliance (WAI-SR), and adherence parameters.

Measure	pre-treatment (T1)		mid-treatment (T2)		post-treatment (T3)		follow-up (T4)	
	guided	unguided	guided	unguided	guided	unguided	guided	unguided
	M (SD)	M (SD)	M (SD)	M (SD)	M (SD)	M (SD)	M (SD)	M (SD)
**BDI-II**	30.09 (9.18)	30.54 (8.53)	20.71 (6.98)	22.51 (7.83)	16.61 (9.55)	18.49 (8.88)	23.86 (9.25)	24.46 (8.41)
WAI-SR								
bond			2.94 (1.34)	2.76 (1.28)	3.67 (0.99)	3.30 (0.99)		
task			2.38 (1.10)	2.36 (0.95)	3.43 (0.81)	3.16 (0.77)		
goal			2.65 (1.19)	2.63 (1.11)	3.52 (0.93)	3.23 (0.92)		
total			2.66 (1.12)	2.58 (1.04)	3.54 (0.83)	3.23 (0.79)		
Adherence parameters								
module progress, M (SD)					9.43 (2.33)	9.28 (2.35)		
Main course completion rate N (%)					130 (86.1 %)	124 (82.7 %)		

Pre-treatment (T1); mid-treatment (T2) = six weeks after pre-treatment; post-treatment (T3) = twelve weeks after pre-treatment; follow-up (T4) = twenty four weeks after pre-treatment. BDI, Beck Depression Inventory; WAI-SR, Working Alliance Inventory – Short Revised. Data reported for the ITT sample.

### 3.4 Working alliance levels

**Figure 2:**
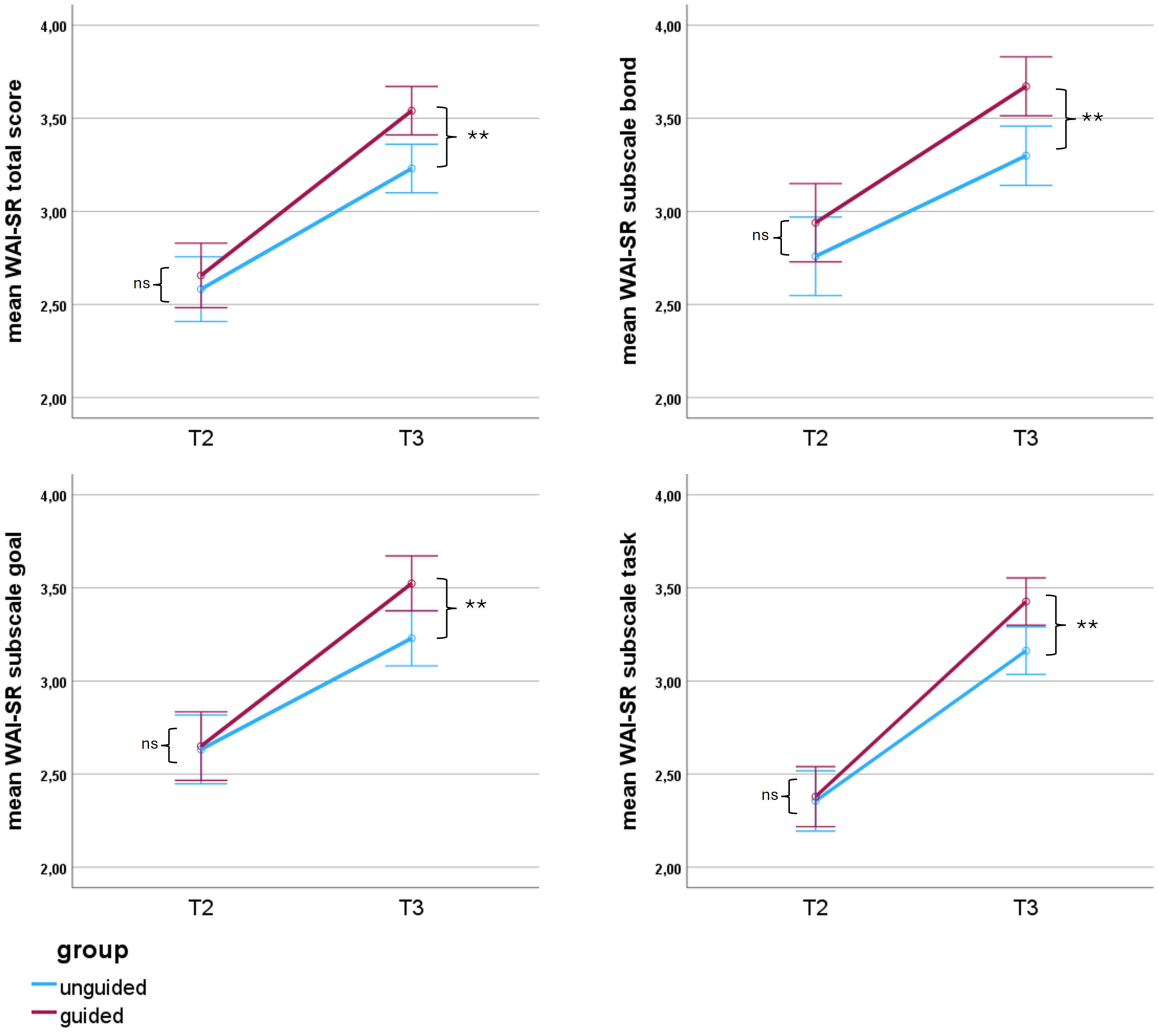
Trajectories of the working alliance (total WAI-SR scores). Notes: WAI-SR, Working alliance inventory -short revised; mid-treatment (T2) = six weeks after pre-treatment; post-treatment (T3) = twelve weeks after pre-treatment; Error-bars indicate 95% CI; Y-scale range adjusted for better visibility. Range of the Working alliance score originally from 0 to 5. *Post-hoc* Bonferroni corrected group difference at T2 and T3: ns, not significant p > 0.05; ** p≤ 0.01.

Trajectories of the total- and subscale WAI-SR scores for both groups are depicted in **Figure 2**. Descriptive results on means and standard deviations of the WAI-SR scores are reported in **Table 2**. A repeated measures ANOVA was conducted to evaluate the effect of group, time and time*group interaction for the WAI-SR scores across the two assessments at mid- and post-treatment. For the total scores significant effects were found for the factor *group* (F 1, 299 = 5.76, p = .017) and *time* (F 1, 299 = 102.18, p <.001), while group*time (F 1, 299 = 2.44, p = .12) was not significant. With regard to the subscales of the WAI-SR, all three subscales, namely *goal*, *bond* and *task* showed a significant effect of *time* but no significant effect of group*time. Only the subscale *bond* showed a significant effect of *group* (p = .004; **Appendix Table 1**). *Post-hoc* pairwise comparisons with Bonferroni-correction revealed no significant differences between the groups at mid-treatment for both total- und subscale scores (all p >.05), but significantly higher total- and subscale scores at post-treatment in the guided compared to the unguided group (all p <.001; **Appendix Tables 2**, **3**). **Table 3** shows within group effect sizes for T2-T3 as well as between-group effect sizes for T2 and T3 for the WAI-SR total- and subscale scores reported as Cohen’s d and 95%-Confidence intervals. Both intervention groups showed an increase in the total und subscale WAI-SR scores from mid- to post-treatment with small to medium effect sizes (Cohen’s d = -0.33 to -0.79). At post-treatment the guided group showed higher total- and subscale WAI-SR scores than the unguided group with small effect sizes (d = -0.32 to -0.38).

**Table 3:** Within and between group effect sizes of the working alliance scores (Cohen’s d).

WAI-SR Scale/Group	Within group effect-sizes		Between group effect-sizes			
	T2-T3		vs. unguided			
			T2		T3	
	d	95%CI	d	95%CI	d	95%CI
total						
guided	-0.65	-0.82; -0.48	-0.07	-0.30; 0.17	-0,38	-0.63; -0.17
unguided	-0.51	-0.67; -0.34				
bond						
guided	-0.45	-0.62; -0.28	-0.14	-0.37; 0.09	-0,38	-0.60; -0.13
unguided	-0.33	-0.50; -0.17				
task						
guided	-0.79	-1.00; -0.61	-0.02	-0.24; 0.22	-0,33	-0.58; -0.09
unguided	-0.73	-0.91; -0.55				
goal						
guided	-0.61	-0.78; -0.44	-0.02	-0.25; 0.21	-0,32	-0.55; -0.09
unguided	-0.42	-0.58; -0.25				

WAI-SR, Working alliance inventory -short revised; total, total WAI-SR score; bond, subscale bond of the WAI-SR; task, subscale task of the WAI-SR; goal, subscale goal of the WAI-SR; T2, mid-treatment; T3, post-treatment; d, effect size Cohen’s d.

### 3.5 Associations between working alliance, change in depressive symptoms and adherence parameters

Partial correlation analyses with WAI-SR scores at mid-treatment (T2) did not reveal significant correlations with change of depressive symptoms at post-treatment (Δ BDI-II T1-T3), at follow-up (Δ BDI-II T1-T4) or with *module progress* in neither group (**Appendix Table 3**). For total WAI-SR scores at post-treatment (**Table 4**), partial correlations indicated a significant positive correlation with change of depressive symptoms at post-treatment in the guided group (correlation coefficient r = .25), while in the unguided group this was the case only for the subscale *task* (r = .18). WAI-SR scores at post-treatment and change of depressive symptoms at follow-up were significantly correlated in both groups (guided: r = .25; unguided: r = .17). In the unguided group this was mainly driven by the subscale task (r = .24). WAI-SR scores at post-treatment and *module progress* were significantly correlated in both intervention groups (guided: r = .28; unguided: r = .25) (**Table 4**). Binary logistic regression analyses (**Appendix Table 4**) revealed no significant association of mid-treatment WAI-SR scores and likelihood of *course completion*. Higher total WAI-SR scores at post-treatment were associated with an increased likelihood of *course completion* in both intervention groups (guided: OR = 2.15; 95%CI [1.23; 3.74]; unguided OR = 2.79; 95%CI [1.46; 5.32]). In the unguided group this was mainly driven by the subscale *task* (OR= 3.04; 95%CI [1.57; 5.89]) (**Appendix Table 4**).

**Table 4:** Partial correlations between the total score and subscales of the working alliance (WAI-SR) at post-treatment (T3) with outcomes at post-treatment and follow-up as well as with the adherence parameter module progress.

WAI-SR score	post-treatment Δ BDI-II (T1-T3)				follow-up Δ BDI-II (T1-T4)				module progress			
	T2		T3		T2		T3		T2		T3	
	guided	unguided	guided	unguided	guided	unguided	guided	unguided	guided	unguided	guided	unguided
total	.02	.12	.25**	.15	.01	-.13	.25**	.17*	-.11	.04	.28***	.25**
bond	.03	.09	.18*	.12	.00	-.14	.23**	.13	-.13	.02	.26**	.25**
task	.00	.15	.29***	.18*	-.04	-.08	.23**	.24**	-.06	.04	.28***	.22**
goal	.02	.09	.22**	.11	.07	-.12	.22**	.17*	-.12	.06	.22**	.19*

* p <.05. ** p <.01.*** p <.001. Results are corrected for BDI-II at pre-treatment (T1).

### 3.6 Mediation analyses

Model 1 yielded no significant direct or indirect effects of guidance on post-treatment outcomes through the working alliance at mid-treatment. As a side finding, the analysis indicated that completion of more modules was linked to a greater reduction in depressive symptoms at post-treatment. Model 2 revealed a significant indirect positive effect of guidance on symptom reduction at follow-up mediated by the working alliance at post-treatment (Path 1: a1*b1 = 0.59, SE = 0.28, 95%CI: [0.14; 1.22]) and a second positive indirect effect through the link between working alliance at post-treatment and *module progress* (Path 3: a1*d1*b1 = 0.15, SE = 0.078, 95%CI: [0.04; 0.34]). There was no independent indirect effect through *module progress* alone (Path 2: a2*b2 = -0.06, SE = 0.18, 95%CI: [-0.42; 0.30]). No significant direct or total effects were observed. Indirect effects remained significant when the model was additionally controlled for previous change in depressive symptoms (Path 1: a1*b1 = 0.47, SE = 0.24, 95%CI: [0.07; 1.02]; Path 3: a1*d1*b1 = 0.09, SE = 0.06, 95%CI: [0.01; 0.22]) (**Table 5**).

**Table 5:** Serial mediation models with working alliance and module progress mediating the relationship between guidance and outcome at post-treatment (model1) and follow-up (model2).

Model 1	b	SE	95%CI		p
			Lower	Upper	
c: total effect	1.858	1.747	-0.235	3.951	.0817
direct effects					
c': Guidance → Δ BDI-II T1-T3	1.623	1.012	-0.367	3.615	.110
a1: Guidance → Working alliance (T2)	0.072	0.125	-0.174	0.317	.574
a2: Guidance → module progress	0.152	0.269	-0.378	0.682	.572
b1: Working alliance (T2) → Δ BDI-II T1-T3	0.739	0.469	-0.185	1.662	.116
b2: module progress → Δ BDI-II T1-T3	1.236	0.218	0.801	1.662	<.001
d1: Working alliance (T2) → module progress	-0.075	0.125	-0.321	0.170	.546
indirect effects*					
Path1: Guidance → Working alliance (T2) → Δ BDI-II T1-T3	0.053	0.108	-0.146	0.304	
Path2: Guidance → module progress → Δ BDI-II T1-T3	0.188	0.343	-0.464	0.903	
Path3: Guidance → Working alliance (T2) → module progress → Δ BDI-II T1-T3	-0.007	0.023	-0.066	0.034	
Model 2					
c: total effect	0.554	1.016	-1.145	2.553	0.586
direct effects					
c': Guidance → Δ BDI-II T1-T4	-0.135	0.996	-2.096	1.825	.892
a1: Guidance → Working alliance (T3)	0.308	0.094	0.123	0.492	.001
a2: Guidance → module progress	-0.085	.0265	-0.606	0.435	.748
b1: Working alliance (T3) → Δ BDI-II T1-T4	1.922	0.627	0.687	3.156	.002
b2: module progress → Δ BDI-II T1-T4	0.666	0.219	.236	1.096	.003
d1: Working alliance (T3)→ module progress	0.754	0.161	0.438	1.070	<.001
indirect effects*					
Path1: Guidance → Working alliance (T3) → Δ BDI-II T1-T4	0.591	0.280	0.138	1.222	
Path2: Guidance → module progress → Δ BDI-II T1-T4	-0.057	0.181	-0.417	0.339	
Path3: Guidance → Working alliance (T3) → module progress → Δ BDI-II T1-T4	0.154	0.078	0.037	0.339	

SE, standard error. * indirect effects based on bootstrapping with 5000 samples to construct a 95% confidence interval of mediating effects. Point estimates of indirect effects are considered statistically significant if zero is not included in the 95% confidence interval. → indicates the direction of influence between variables.

## 4 Discussion

In the present study, we examined the trajectories of the working alliance and its link to guidance during and after treatment of depressive symptoms with the IBI Selfapy as part of a secondary analysis of a previously published RCT (20). We investigated the working alliance in both a guided and unguided IBI, building on a conceptual framework suggesting that an alliance can be formed with both the human and technological aspects of an intervention (11, 34). Supporting this concept, analyses indicated that the IBI successfully fostered a robust working alliance in both intervention groups. However, while the alliance ratings were similar at mid-treatment, they were significantly higher in the guided group compared to the unguided group at post-treatment, with a small effect size. Due to the lack of normative data to interpret the WAI-SR, Jasper et al. (35) have suggested a categorization based on a simple tripartite division of the score range into low (score: 1.00–2.44), medium (score: 2.45–3.44), and high (score: 3.45–5.00). Following this categorization, the mean WAI-SR scores at post-treatment reported here, correspond to a high working alliance in the guided group and a medium working alliance in the unguided group. Post-treatment WAI-SR total scores corresponded to 71% of the maximum score in the guided group and 65% in the unguided group. These results are in line with results by Bur et al. (10), who compared ratings of the working alliance of a guided and unguided group during an 8-week IBI for depressive symptoms. At post-treatment they reported working alliance ratings of 72% of the maximum score for the guided group and 63% in the unguided group. Similar ratings have also been reported for other IBIs for depression utilizing the Working Alliance Inventory (WAI) (36) or the Working Alliance Inventory for guided Internet interventions (WAI‐I) (37). These results are also in line with what can be usually found in f2f treatments (25, 38) and support the previously stated conclusion that a working alliance in IBIs can be established without f2f contact (13, 16, 39). Based on our dataset, guidance may enhance the working alliance, although this effect was not yet evident at mid-treatment. This finding is somewhat at odds with a previous study (10), which reported significantly higher alliance ratings in a guided (personalized support via email) compared to unguided intervention even in early treatment. In this context, it is important to note, that different modalities and intensities of guidance could yield diverse effects on the working alliance and may thus not be directly comparable (11). In comparison to most studies involving some form of guidance, the intensity of guidance in our study was relatively high, with an average of 8 calls per participant with a mean duration of 22 minutes each. For example, in their meta-analysis including 47 studies providing human therapeutic guidance, Moshe et al. (2) reported an average guidance duration of 80.93 minutes (SD = 38.44) across the entire intervention. Furthermore, the unguided group had the option to contact study personnel via chat for technical questions. As previously reported (24), analysis of the chat function usage data revealed no significant difference between the groups in the median number of messages sent. However, the unguided group wrote significantly longer messages compared to the guided group, possibly compensating for their lack of access to phone calls. These factors should be considered when interpreting the data. For one, the availability of human contact in both treatment arms may explain in parts the large effect sizes in reducing depressive symptoms in both intervention groups (20) and the comparably low drop-out rate in the unguided group. For another the variations in human contact might not have been enough to elicit more substantial differences between the guided and unguided groups both with regards to outcomes as well as scores of the working alliance. In the present study, guidance content was focused on reflecting on exercises and addressing topics such as personal resources and behavioral activation. Similar emphases of guidance on elements that are covered by the task and goal subscales of the working alliance have also been reported for other IBIs (40). The goal and task elements of the working alliance are thought to be closely tied to beliefs and expectations about treatment effectiveness (41). Conceptually, it seems plausible that interaction with a real therapist, who reinforces these beliefs and expectations and validates personal difficulties in implementation, would lead to higher ratings on the goal and task subscales. Concurrently, joint reflection on goals and tasks with a therapist may inherently strengthen the sense of bond. Consequently, the generally higher ratings across all subscales through guidance could be viewed as a joint effect.

In the present analyses, post-treatment working alliance scores were positively associated with symptom reduction both at post-treatment and follow-up in both groups, though the strength of this association varied. In the guided group, the association was generally stronger and was mainly driven by the subscale tasks at post-treatment and about equally by all subscales at follow-up. In the unguided group, the positive association was primarily driven by the subscale task at both time points. These findings align with previous research suggesting that agreement on tasks and goals may be of particular importance in guided IBIs (13) and bCBT (15). They further indicate that this agreement, especially concerning tasks, might be even more crucial in unguided IBIs. No significant associations were found between working alliance ratings at mid-treatment and symptom reduction in either group, suggesting limited predictive value at this stage. In this regard, future investigations might benefit from incorporating therapist-rated working alliance, as this approach has been reported to better predict subsequent changes in depression symptoms in bCBT (19, 42). Advances in machine learning could potentially even enable the assessment of the alliance without requiring therapist ratings (43) making it a promising approach particularly for unguided IBIs. While there are reports of ratings of the working alliance to be associated with outcomes and adherence as early as two weeks after pre-treatment in an 8-week IBI (10), it is an often reported phenomenon that correlations of the working alliance with outcomes are greater when the alliance is measured toward the end of the IBI (17). In this regard it has been argued that a strong working alliance could also be seen as an effect than a cause of successful treatment (32, 36, 44). Others have suggested a model of reciprocal influence of working alliance and outcome (45). Here, we hypothesized a mediating effect of the working alliance and adherence in the relationship between guidance and outcomes. Our results support this hypothesis to some extent in that we found evidence for a mediating effect of post-treatment working alliance on the relationship between guidance and outcomes at follow-up. Moreover, guidance was sequentially associated with working alliance in the first step, which further positively influenced adherence, which, in turn, was linked to a greater reduction in depressive symptoms at follow-up. Importantly, these mediating effects remained significant when controlling for prior symptom improvement, supporting a reciprocal influence model of the relationship between working alliance and symptom change (46). These findings also align with prior research suggesting that the working alliance may play an important role with regard to long-term outcomes in guided IBIs (11, 47). Somewhat unexpected, there was no significant mediating effect of adherence per se. This could be due to the chosen adherence parameter *module progress*, which arguably does not adequately reflect the overall adherence. Bur et al. (10) reported independent effects of both adherence and working alliance, each of which explained a part of the variance in the guidance effect. Fuhr et al. (29) reported that the prediction of a reduction in depressive symptoms based on adherence after completion of an IBI could be explained mainly by receiving guidance. Of note, all studies in this context (including our own) used different approaches to quantify adherence based on interaction with the IBI within sessions. A more extensive quantification of adherence that captures not only engagement within but also between therapeutic sessions could be insightful here. Finally, while improved adherence may account for some of the positive effects of a strong working alliance, recent studies also point to other interrelated factors like emotion regulation (48, 49).

The findings presented here may offer insights that could inform the ongoing development of IBIs for treating depression. With regard to the working alliance, it appears that emphasizing the elements goals and tasks may at least in parts offset the absence of f2f bonding opportunities in IBIs. In particular an agreement on tasks seems important with regard to short and long-term outcomes. In light of this, treatment providers may consider adjusting IBIs to enhance participants’ alignment with tasks by incorporating e.g. more possibilities to collaborative decision-making into therapy content, and agreement on personalized short- and long-term goals. The importance of personalized content opposed to a “generic program” has also been stressed by a recent qualitative study (50). With regard to the bond subscale it will be crucial to examine the impact that advancements in areas such as affective computing will have in this context (51). Seen that a strong working alliance might be also of importance with regard to long term outcomes, the integration of follow-up modules could serve to maintain the sense of alliance, thus mitigating the sudden cessation of the therapeutic relationship. Additional therapeutic guidance may enhance the working alliance and thereby increase adherence and improve outcomes. At the same time our analyses suggested that both unguided and guided groups achieved a similar reduction in depressive symptoms, even though the unguided group had lower post-treatment ratings of the working alliance. This could suggest that particularly the success of unguided IBIs may additionally hinge on other factors, that were not measured here, such as a sense of increased self-efficacy derived from independently navigating the intervention or the advantages of anonymity. Nevertheless, it is important to note that in real-world routine use of IBIs outside of controlled studies, the working alliance and its link to guidance and adherence may have a notably greater impact. With the absence of screening procedures and regular assessments, personalized contact in unguided pure self-help IBIs in routine settings could be reduced to a minimum compared to controlled studies (efficacy studies). As a result, the positive influence of guidance on working alliance, adherence and consecutively on outcomes may become more evident. Indeed, findings from a meta-analysis of studies conducted in routine settings (effectiveness studies) underscore the importance of guidance, particularly highlighting the advantages of therapeutic guidance over mere technical guidance (2). This also aligns with a qualitative study which found that a lack of guidance was a primary reason cited for participants dropping out of the program (50).

## 5 Limitations

The results presented here should be interpreted in the context of some limitations. First, like many RCTs in this field, we share the potential limitation that our sample may have been composed of participants who were particularly motivated to try an IBI, which may have influenced ratings and thus may limit generalizability. Second, a further limitation regarding generalizability must be made with respect to the German population studied here, insofar as we contribute to the previously criticized overrepresentation of the Northern European region in current research in this field (52). Third, the dismantling approach used here allowed us to explicitly test for the potential impact of guidance via weekly phone calls. It should be noted however that different modalities of guidance may have different effects on the working alliance (11). Future research should examine whether the associations reported here vary between different guidance modalities. Fourth, while our study follows common definitions by classifying the treatment arms as guided and unguided IBI [e.g., (4)], the intensity of guidance (averaging 8 calls at 22 minutes each) was relatively high compared to other studies (2), and approaches the about 7 f2f sessions averaging about 50 minutes each reported for blended therapy formats (53). This high intensity may limit comparability with other guided interventions that use less intensive guidance. Unlike typical blended therapy, guidance in this study did not involve f2f sessions and therapists merely provided clarification and reinforcement of the preexisting therapeutic content of the IBI, which was designed as a self-help intervention (54). Participants in the unguided group had access to a chat function for non-content-related questions, which might have influenced their perception of the working alliance, given that even technical guidance may improve outcomes and adherence (2). Therefore, the unguided group might also be construed as having “technical guidance on demand.” These remarks highlight the challenges of using inconsistent terminology in IBI research and the need for more standardized definitions (55). Fifth, the WAI-SR, used to assess the working alliance in our study, was originally designed for traditional f2f psychotherapy. We assumed that the interpersonal alliance concept (human-to-human) would be transferable to IBIs (human-to-software) and their combination with human contact (human-to-software+human), and that participants would intuitively interpret the questions in this context (56, 57). While our data support these assumptions to some extent, the WAI-SR does not differentiate whether responses reflect interactions with the program, the therapist during phone calls (in the guided group), chat support, human contact during screening, or general evaluation of the treatment provider. Consequently, the perceived alliance may be influenced by a blend of these elements rather than any single component. Future research should explore whether our findings can be replicated and improved using conceptually adapted tools, such as the WAI-Tech (58) and WAI-I (37), and methods that e.g. separately evaluate the alliance with both the program and the therapist (59).

## 6 Conclusion

Consistent with the primary study, the present secondary analysis revealed no significant direct effect of guidance on outcomes. However, findings of the current analysis suggest that guidance may be beneficial by enhancing the working alliance and consecutively adherence. The advantages of a strong working alliance could prove particularly significant in the everyday implementation of both guided and unguided IBIs beyond controlled research settings, underscoring the necessity for additional research in this context. Future research should also aim to identify subgroups of patients and contexts where the interplay between guidance and a strong working alliance is particularly beneficial. For example, this could include patients stratified by the severity of depression (4). At the same time further research is necessary to better understand factors that enhance the working alliance in unguided IBIs and identify contexts in which unguided IBIs might be preferable due to unique strengths such as perceiving them as more anonymous, less judgemental and more convenient (34).

## Data Availability

The raw data supporting the conclusions of this article will be made available by the authors, without undue reservation.
