# Supplementary material for: Working alliance and its link to guidance in an internet-based intervention for depressive disorders: a secondary analysis of a randomized controlled trial
Source: Front Psychiatry. 2024 Sep 11;15:1448823. doi: 10.3389/fpsyt.2024.1448823 (PMC11422127; doi:10.3389/fpsyt.2024.1448823)
Supplement: Supplementary file 1 [file Table1.docx]

# Appendix/Supplementary Material

Appendix Table 1. Results of repeated measure ANOVA on WAI-SR total scores and subscales

| WAI-SR Scale/factor | |  |
| --- | --- | --- |
|  | F | p |
| **total** |  |  |
| group | 5.76 | .017 |
| time | 102.18 | <.001 |
| group*time | 2.44 | .12 |
| **bond** |  |  |
| group | 8.34 | .004 |
| time | 46.21 | <.001 |
| group*time | 1.06 | .305 |
| **task** |  |  |
| group | 3.45 | .064 |
| time | 173.29 | <.001 |
| group*time | 2.92 | .089 |
| **goal** |  |  |
| group | 3.67 | .075 |
| time | 79.47 | <.001 |
| group*time | 2.82 | .094 |

Appendix Table 2. Post-hoc bonferroni corrected p-values of pairwise comparisons of WAI-SR total scores and subscales at mid-treatment (T2).

| WAI-SR score T2/Group | vs. unguided | |
| --- | --- | --- |
|  | p | 95%CI |
| **total** |  |  |
| guided | .56 | -.17 - .32 |
| **bond** |  |  |
| guided | .234 | -.12 - .48 |
| **task** |  |  |
| guided | .84 | -.21 - .25 |
| **goal** |  |  |
| guided | .89 | -.24 - .28 |

Appendix Table 3. Post-hoc bonferroni corrected p-values of pairwise comparisons of WAI-SR total scores and subscales at post-treatment (T3).

| WAI-SR score T3/Group | vs. unguided | |
| --- | --- | --- |
|  | p | 95%CI |
| **total** |  |  |
| guided | .001 | .13 - .46 |
| **bond** |  |  |
| guided | .001 | .15 - .60 |
| **task** |  |  |
| guided | .004 | .08 - .44 |
| **goal** |  |  |
| guided | .006 | .06 - .50 |

Appendix Table 4. Odds-ratios for the likelihood of course completion (six obligatory modules) based on working alliance scores at mid- and post-treatment (T2,T3)

| WAI-SR score | T2 | | | |  | T3 | | | |
| --- | --- | --- | --- | --- | --- | --- | --- | --- | --- |
|  |  |  |  |  |  |  |  |  |  |
|  | guided | | unguided | |  | guided |  | unguided | |
|  | OR | 95%-CI | OR | 95%-CI |  | OR | 95%-CI | OR | 95%-CI |
| total | 0.90 | 0.59; 1.37 | 0.90 | 0.75; 1.69 |  | 2.15** | 1.23; 3.74 | 2.79** | 1.46; 5.32 |
| bond | 0.90 | 0.63; 1.28 | 0.90 | 0.74; 1.43 |  | 1.95** | 1.23; 3.10 | 1.91** | 1.21; 3.03 |
| task | 0.99 | 0.64; 1.53 | 0.99 | 0.80; 2.04 |  | 2.29** | 1.29; 4.07 | 3.04*** | 1.57; 5.89 |
| goal | 0.87 | 0.59; 1.29 | 0.87 | 0.75; 1.61 |  | 1.58 | 0.96; 2.58 | 1.79* | 1.10; 2.92 |

Notes: * p < .05. ** p < .01.*** p < .001.
